# Policy and the Transparency of Values in Science

**DOI:** 10.1289/ehp.1408936

**Published:** 2014-11-01

**Authors:** David H. Schwartz

**Affiliations:** Innovative Science Solutions, LLC, Morristown, New Jersey, USA, E-mail: schwartz@innovativescience.net

I read with interest the article “Science Policy, and the Transparency of Values” (Elliott and Resnik 2014) and wanted to make a few comments and observations.

First, the authors noted that different levels of scientific evidence are necessary depending on the venue in which that evidence is going to be used: High standards of evidence are necessary to infer causal relationships, with weaker standards of evidence required to protect the public from perceived risks. Although I agree that different venues or contexts require different standards of evidence, it is important to emphasize that the actual scientific evidence remains constant. For example, anecdotal evidence is the weakest level of scientific evidence on the hierarchy, regardless of the context in which it is being employed. There might be a legitimate rationale to relax the level of evidence required to inform policy within a given context, but that does not change the nature of the evidence itself. If we rely on “weaker” standards of evidence to protect the public, the basis of the decision and the nature of the evidence relied upon should be made transparent. This is not always the case.

Second, the authors combined tort law and chemical regulations as examples in which weaker standards of evidence can suffice to inform policy. I disagree with treating these two venues as equivalent. Rather, tort law should be part of the first venue: inferring causal relationships. After all, the goal of a civil tort is to prove that a chemical causes the alleged injury. Legal tort cases are not intended to be theoretical or precautionary exercises.

Third, in the “Discussion” of their paper, Elliott and Resnik (2014) pointed out that when authors have ties to regulated industries, these relationships could serve to influence the interpretation of findings and the conclusions drawn. Fair enough. But Elliott and Resnik failed to emphasize that financial ties to industry are only one type of conflict of interest. For example, ideological ties to advocacy organizations are another strong source of potential conflict of interest that could adversely influence the use of science in the interest of public policy. When using science to inform policy, transparency is critical. However, this transparency should include not only financial ties to industry but also ties to advocacy organizations and other strongly held points of view.

Finally, it seems incomplete to assess the role of science in public policy without a discussion of the importance of evaluating risk–benefit relationships. Clearly, society is willing to tolerate health risks from certain chemical exposures when those risks are deemed to be outweighed by the benefits. This risk–benefit assessment is made every time a new drug is considered for approval. In this context, policy makers are willing to tolerate great risk if the benefits of a pharmaceutical agent are deemed to outweigh the risks (and if the appropriate warnings are made). Conversely, policy makers are appropriately unwilling to tolerate health risks when the benefits are minimal or inadequately defined. Characterizing the risk–benefit relationship is critical to setting rational and appropriate public policy. Any discussion of the role of science in this endeavor is inadequately served without discussing these important relationships.

I am a scientific consultant who works with commercial manufacturers to help them understand the science related to their products. I have a deep and enduring respect for rigorous scientific inquiry using the best and most appropriate scientific principles. These principles and methodologies are clearly defined and should be carefully and systematically applied before using scientific findings to influence policy (e.g., Rooney et al. 2014). The nature of the available scientific evidence does not change based on the context in which it is applied. It is ultimately the job of policy makers to review the scientific evidence rigorously and systematically. Policy should then be set according to a set of rules that is consistent, rational, and transparent; free of conflict; and informed by the available science.
